# Supplementary figures and images for: Phylodynamics of a regional SARS-CoV-2 rapid spreading event in Colorado in late 2020
Source: PLoS One. 2022 Oct 4;17(10):e0274050. doi: 10.1371/journal.pone.0274050 (PMC9531818; doi:10.1371/journal.pone.0274050)

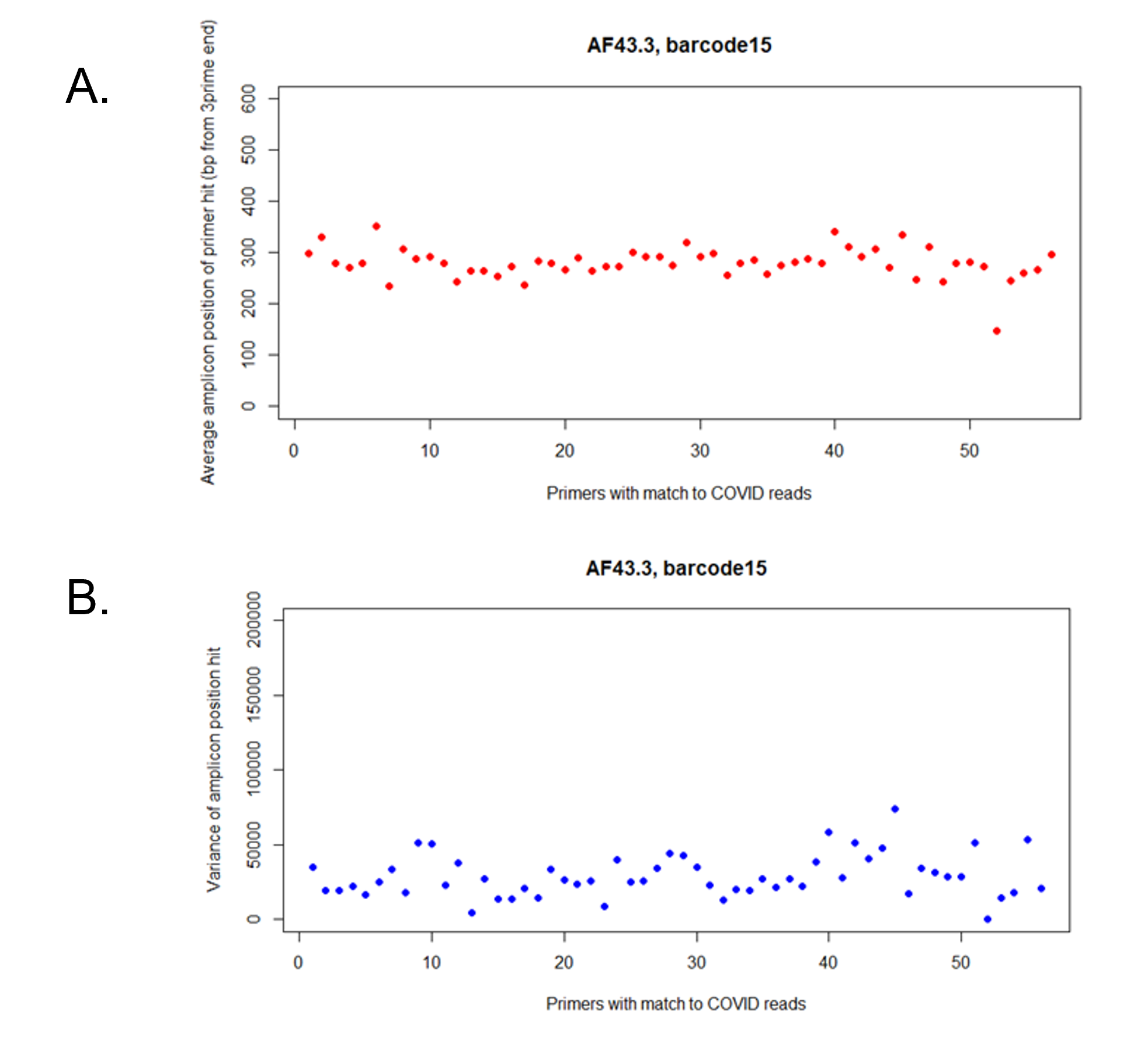

Supplement: S1 Fig — 1A shows the average position along the y axis, primer number along the x axis. 1B shows the variance calculated for the distribution of matching positions for each primer. (TIF) [file pone.0274050.s001.tif]

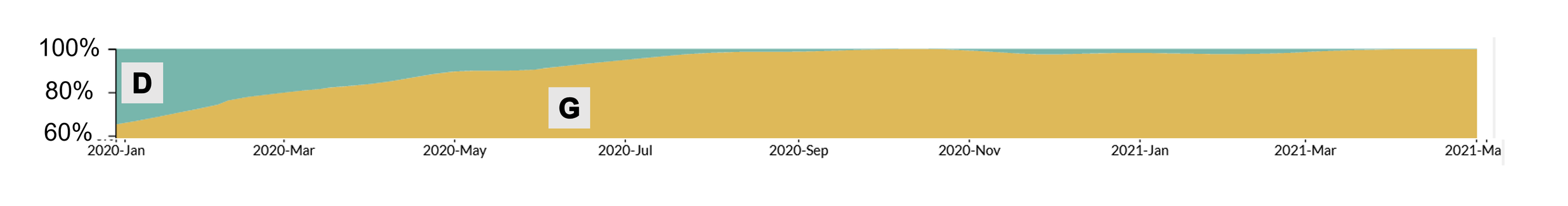

Supplement: S2 Fig — Gold indicates frequency of ‘G’ substitution over time, green indicates frequency of ‘D’ substitution over time. (TIF) [file pone.0274050.s002.tif]

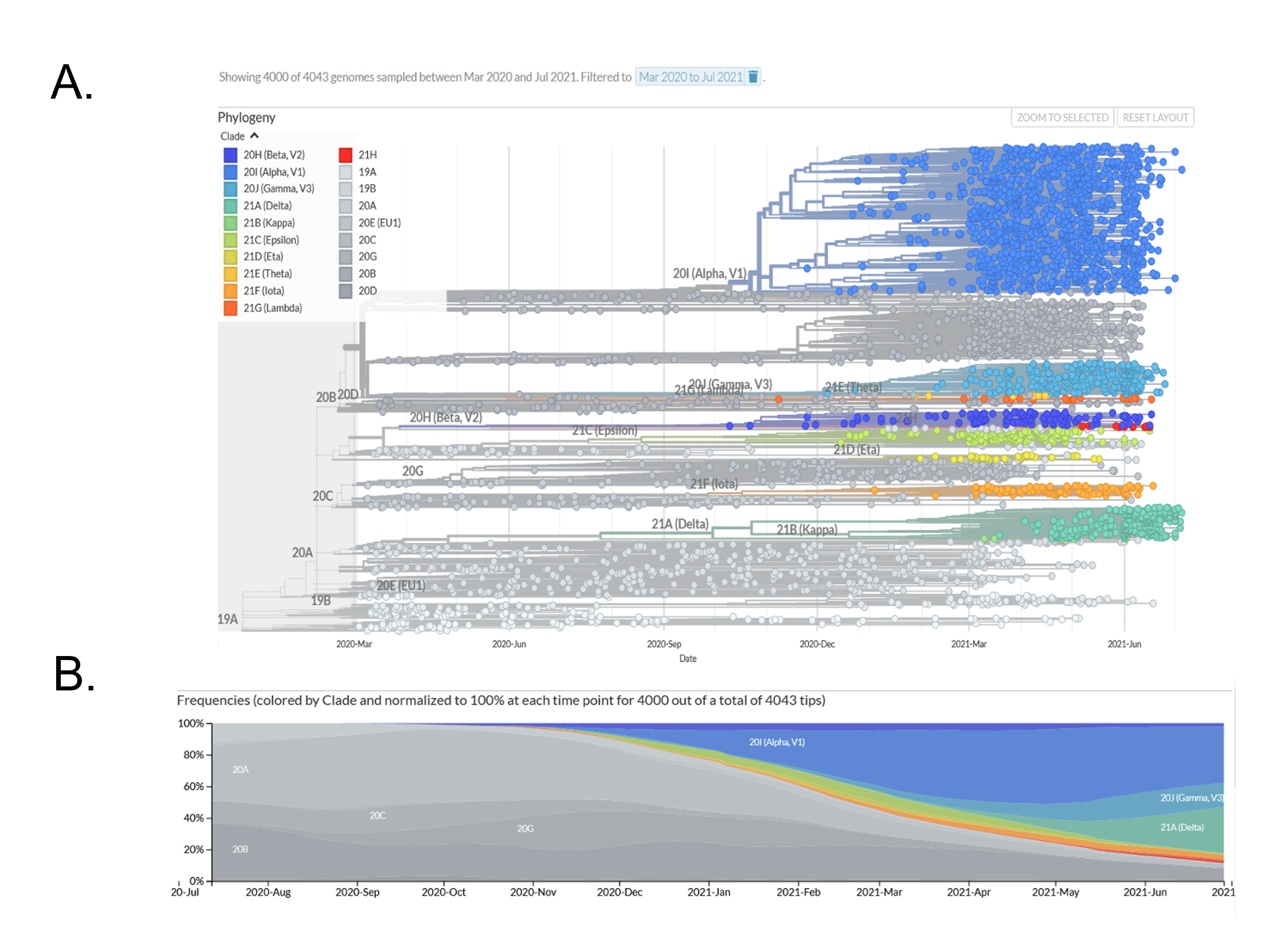

Supplement: S3 Fig — A) Phylogenetic relationships of strains across the continent. B) Strain relative prevalence between August 2020 and July 2021. Date accessed July 12, 2021. (TIF) [file pone.0274050.s003.tif]

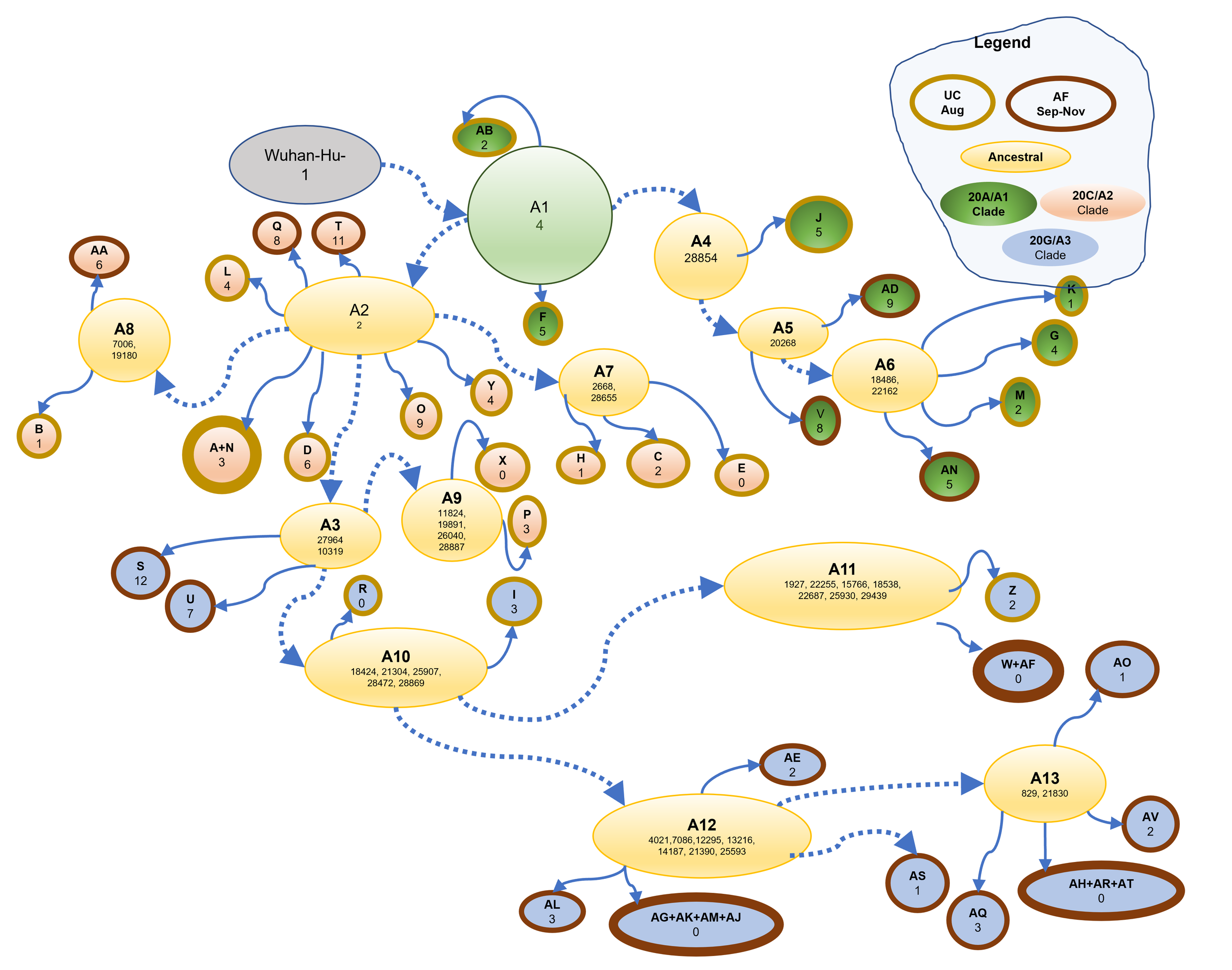

Supplement: S4 Fig — Each tip indicates the genome letter identifier and the number of mutations away from its most recent ancestral node. Ancestral nodes contain the name of the node (A1-A13) and the position of lineage-defining mutations which are inherited by all downstream lineages. Node and tip coloring described in legend. (TIF) [file pone.0274050.s004.tif]

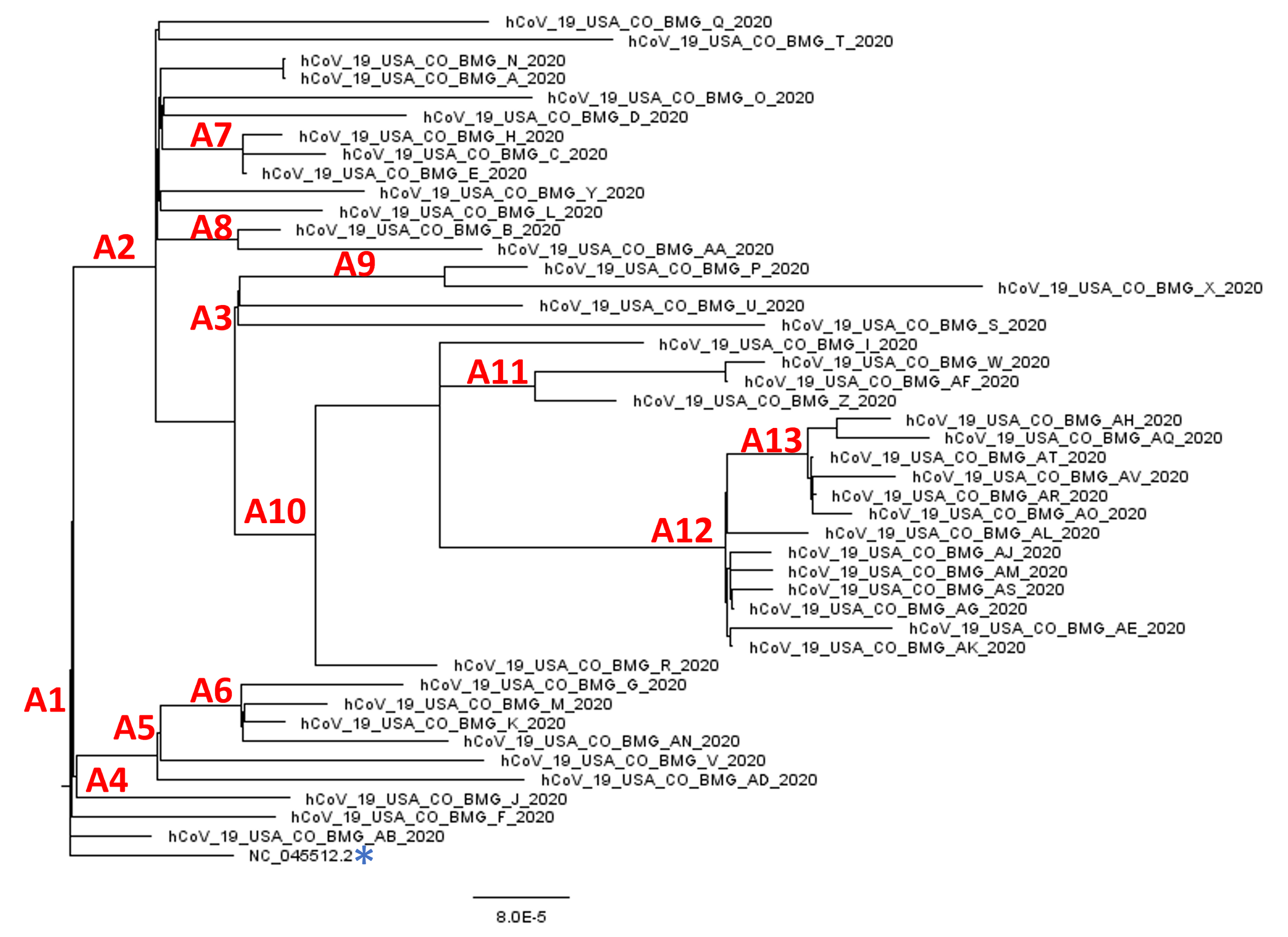

Supplement: S5 Fig — Genomes were aligned with Mugsy and the consensus tree was generated under the Jukes-Cantor model using IQTree. Tip labels indicate the sample names as identifiable in GISAID. Red branch labels indicate the major phylogroups described in this paper. Blue asterisk indicates the Wuhan-Hu-1 genome outgroup, NC_045512.2. (TIF) [file pone.0274050.s005.tif]

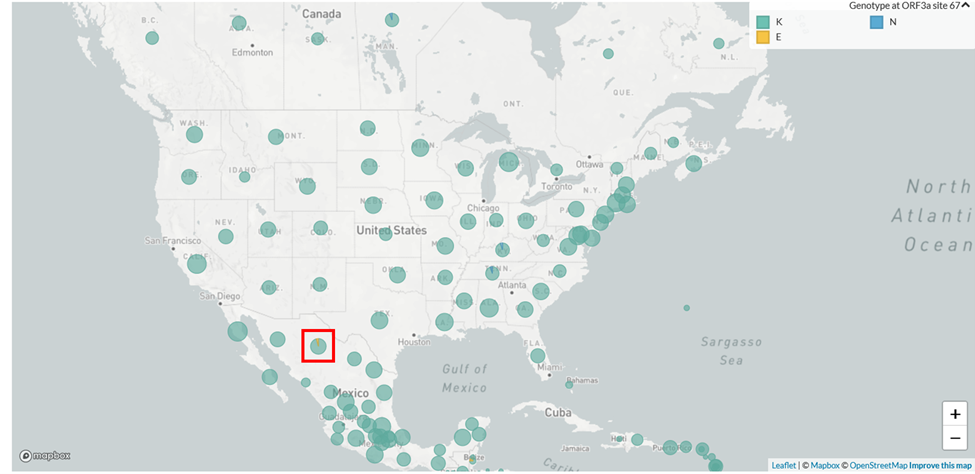

Supplement: S6 Fig — The only other documented instance of this variant in the NextStrain repository occurs in Northern Mexico. Map image downloaded from NextStrain, which uses OpenStreetMap®. OpenStreetMap® is open data, licensed under the Open Data Commons Open Database License (ODbL) by the OpenStreetMap Foundation (OSMF). (TIF) [file pone.0274050.s006.tif]
