# Supplementary material for: Phylodynamics of a regional SARS-CoV-2 rapid spreading event in Colorado in late 2020
Source: PLoS One. 2022 Oct 4;17(10):e0274050. doi: 10.1371/journal.pone.0274050 (PMC9531818; doi:10.1371/journal.pone.0274050)
Supplement: S1 File — (DOCX) [file pone.0274050.s007.docx]

EPI_ISL_3841950,

EPI_ISL_3841972,

EPI_ISL_3841951,

EPI_ISL_3841973,

EPI_ISL_3841952,

EPI_ISL_3841974,

EPI_ISL_3841953,

EPI_ISL_3841975,

EPI_ISL_3841990,

EPI_ISL_3841991,

EPI_ISL_3841970,

EPI_ISL_3841971,

EPI_ISL_3841958,

EPI_ISL_3841959,

EPI_ISL_3841954,

EPI_ISL_3841976,

EPI_ISL_3841955,

EPI_ISL_3841977,

EPI_ISL_3841956,

EPI_ISL_3841978,

EPI_ISL_3841957,

EPI_ISL_3841979,

EPI_ISL_3841961,

EPI_ISL_3841983,

EPI_ISL_3841962,

EPI_ISL_3841984,

EPI_ISL_3841963,

EPI_ISL_3841985,

EPI_ISL_3841964,

EPI_ISL_3841986,

EPI_ISL_3841980,

EPI_ISL_3841981,

EPI_ISL_3841960,

EPI_ISL_3841982,

EPI_ISL_3841969,

EPI_ISL_3841948,

EPI_ISL_3841949,

EPI_ISL_3841965,

EPI_ISL_3841987,

EPI_ISL_3841966,

EPI_ISL_3841988,

EPI_ISL_3841967,

EPI_ISL_3841989,

EPI_ISL_3841968
