# Supplementary material for: Phylodynamics of a regional SARS-CoV-2 rapid spreading event in Colorado in late 2020
Source: PLoS One. 2022 Oct 4;17(10):e0274050. doi: 10.1371/journal.pone.0274050 (PMC9531818; doi:10.1371/journal.pone.0274050)
Supplement: Attachment — Submitted filename: ResponseToReviewers_11august22.docx [file pone.0274050.s017.docx]

We thank the reviewer for their thoughtful comments about our manuscript, and as we detail below, we have made changes to the manuscript to address the molecular dating concerns raised by the reviewer.

*Below we address specific comments from the editor and reviewer. For clarity, the reviewer comments are in black, and ours are in blue.*

**Editor comments:**

1. Please ensure that your manuscript meets PLOS ONE's style requirements, including those for file naming. The PLOS ONE style templates can be found at 
<https://journals.plos.org/plosone/s/file?id=wjVg/PLOSOne_formatting_sample_main_body.pdf> and <https://journals.plos.org/plosone/s/file?id=ba62/PLOSOne_formatting_sample_title_authors_affiliations.pdf>

The manuscript style formatting has been revised according to the guidelines.

2. Please state what role the funders took in the study.  If the funders had no role, please state: "The funders had no role in study design, data collection and analysis, decision to publish, or preparation of the manuscript."

Thank you for bringing this matter to our attention; this statement has now been included.

3. Your ethics statement should only appear in the Methods section of your manuscript. If your ethics statement is written in any section besides the Methods, please delete it from any other section.

The ethics statement has been moved to the Methods section.

4. Please include captions for your Supporting Information files at the end of your manuscript, and update any in-text citations to match accordingly. Please see our Supporting Information guidelines for more information: <http://journals.plos.org/plosone/s/supporting-information>.

The captions for Supporting Information are present in the text and at the end of the manuscript (after References), according to the SI guidelines.

**Reviewer comments:**

Reviewer #1: The manuscript by Wade and colleagues is a very interesting piece of work.
Although I am not English native speaker, to my opinion the manuscript is well written in a clear language.

We thank the reviewer for their positive comments.

It describes the phylodynamics of a given lineage that to the Authors likely originated in the local Colorado Springs community and expanded rapidly over the course of two months in an outbreak within the well-controlled environment of the United States Air Force Academy.
Anyhow, the work-flow of analyses contains a big lacking: the molecular dating. I suggest the Author to perform a molecular dating and the estimation of the evolutionary rate. This kind of result will be very useful in order to have a much more complete point of view on the temporally origin and diffusion of the lineage.

We have now performed Bayesian evolutionary modelling of the USAFA SARS-CoV-2 genomes, using their sampling dates from August-November 2020 to inform construction of the phylogenetic tree estimates and inference of divergence rates. This was performed using the Bayesian Evolutionary Analysis Sampling Trees (BEAST) software analysis suite. In doing so, we estimated the divergence of the novel CO Springs variant to a likely origin around October 26, 2020. This agrees with the predictions of the USAFA epidemiological modelling (Sitko et al., 2021), which traced the rapid spreading outbreak back to an event in late October 2020. Our additional modelling and divergence estimate provides further resolution of the related epidemiological data and more robustly supports the conclusions of this manuscript. This new analysis has been included in the revised manuscript.
